# Editorial: “After Industry” the Economic and Social Consequences of Deindustrialization

**DOI:** 10.3389/fsoc.2021.645027

**Published:** 2021-03-18

**Authors:** Jon Warren, Carol Stephenson, Jonathan Wistow

**Affiliations:** ^1^ Department of Sociology, Durham University, Durham, United Kingdom; ^2^ Department of Social Sciences, Northumbria University, Newcastle Upon Tyne, United Kingdom

**Keywords:** class, post-industrial, employment, identity, rerepresentation

Since the 1960’s the UK, Europe and the US have undergone rapid and traumatic changes as major industries have contracted or disappeared altogether—the process of deindustrialization. Employment in manufacturing industries in the UK reduced from 8.9 million in 1966 to just 2.9 million in 2016 (Beatty and Fothergill). The world’s first industrial society is now a post-industrial society, and although the UK is an extreme, it stands as an example for a global phenomenon. Deindustrialization has consequences beyond economic organization and workplaces themselves (Walkerdine and Jimenez, 2012). This matters most for how people construct their own identity. Industries change, develop; adapt and sometimes disappear altogether. However, it is clear that the ways of life, attitudes towards work, family and community; practices and expectations for both men and women, they helped to establish, endure. They remain as “industrial structures of feeling” Williams (1973). For good or ill they persist and that persistence is significant. Understanding “Post-industrial” places is not just a matter of charting economic change. To meaningfully understand we have to explore processes of social and cultural transformation.

The theme of transformation and how ideas about class and identity have changed runs through a number of the contributions to the collection. David Byrne asks how it is possible for social scientists to “do class” in a post-industrial world which is very different to the one in which our core ideas about class were forged. Starting from the premise that interaction between theoretical framings and empirical investigation is at the heart of social science Byrne explores how this can be done and argues that it is essential if we are to make sense of class in a post-industrial world.

Jon Warren approaches the changing nature of class and identity over the period 1969–2019 from a different angle. Reflecting on his personal biography, the biography of North East England and the temporal biography of deindustrialization, this piece uses the lens of Clement and La Frenais well known situation comedy “Whatever happened to the Likely Lads?” (1973) to look at how opportunities for social mobility, and the narratives and expectations that went with them have both changed and, to some extent, endured over the course of his lifetime.

Katy McEwan picks up on the theme of social mobility and what that means in the 21st century. She also offers an insight into how we both conceptualize and research class in a post-industrial setting. Focussing on Teesside’s Ingelby Barwick, the second largest private housing estate in Europe, McEwan explores how the residents of the estate attribute their social mobility and their ability to live on the estate to meritocratic ideas about individual self-reliance, whilst in reality this their lifestyle is often built upon highly precarious economic foundations.

The papers by John Tomaney, Jean Spence, and Carol Stephenson and Fiona MacPherson explore the representation and legacy of past industries in the contemporary era.

Tomaney examines the ongoing importance and vitality of the Durham Miners’ Gala despite the demise of deep coal mining. Drawing on a range of sources he charts the shifting cultural and political meaning of the Gala over time. The Gala, he argues, is an example of an intangible cultural heritage through which knowledge of previous class-based ways of living are reproduced and reimagined.

Spence’s examination of the unrecognized, but accomplished, coal miner-artist Jimmy Kays casts light on the exercise of class-based power in the production, consumption, and range of meaning inscribed within popular mining art: marketability in mining art depends on palatable, often romanticized, representations of mining life in preference to those that connect past and contemporary social and political struggles. As a consequence, Kays, she argues, will always be an ‘outsider’.

Stephenson and MacPherson’s paper explores the potential of cross-disciplinary approaches for community-led creative representation of shared industrial heritage in a post-fishing community. The application of the principals and practices of dialogical discourse, and the sociological observation and analysis of the negotiation of the creative performance provides a new methodological research approach to working with and for divided communities.

The papers by Beatty and Fothergill, Bennett, and Emery are all concerned with place-based consequences of deindustrialization in the UK. In, “The long shadow of job loss”, Beatty and Fothergill draw on their extensive research at the Centre for Regional Economic and Social Research to provide a long view of economic change in Britain's older industrial towns. Their research demonstrates how the trajectories of particular kinds of post-industrial places share characteristics in the current labour market. The title of the paper captures the cumulative effects of deindustrialization in these place. Bennett’s paper is also concerned with the trajectory of place and focuses on life histories in a post-industrial town, Wigan. Through exploring decisions people have made through their working lives a changing landscape is identified through biographical narratives across four generations of families. Changes to employment opportunities across genders and parental influence over career choice emerged as important findings from this study. In, After Coal, Emery explores belonging and alienation in the Nottinghamshire coalfield as a deindustrialized place. Through a range of qualitative methods he draws out the significance of space and place to class-based experiences through intergenerational transmission and shared declarative memories contingent on living through and with deindustrialization in terms of place histories.

This diverse and thought provoking collection of articles explores the economic social and cultural impact of de-industrialization which draw upon a wide range of perspectives whilst utilizing a multiplicity of approaches and methods. In so doing it offers an integrated understanding of the spatial, economic and cultural socioscapes in post-industrial regions and the lives of those who live within them.
